# The application value of the Modified Early Warning Score combined with age and injury site scores in the evaluation of injuries in emergency trauma patients

**DOI:** 10.3389/fpubh.2022.914825

**Published:** 2022-11-23

**Authors:** Qing Li, Yu-Qin Ren, Yu-Fei Qian, Dan-Feng Li

**Affiliations:** Department of Emergency, Nantong First People's Hospital, Nantong, China

**Keywords:** trauma, the Modified Early Warning Score, age, injury site, pre-screening triage

## Abstract

**Objective:**

To explore the application value of the Modified Early Warning Score (MEWS) combined with age and injury site scores in predicting the criticality of emergency trauma patients.

**Methods:**

The traditional MEWS was modified by combining it with age and injury site scores to form a new MEWS combined scoring standard. The clinical data were collected from a total of 372 trauma patients from the emergency department of the Nantong First People's Hospital between June and December 2019. A retrospective analysis was conducted, and the patients were scored using the MEWS combined with age and injury site scores. The patients were grouped according to their prognoses and clinical outcomes. A statistical analysis was conducted based on the ranges of the combined scores, and the results of the combined scores of the different groups were compared.

**Results:**

Among the 372 patients, the average score was 3.68 ± 1.25 points in the survival group, 8.33 ± 2.24 points in the death within 24 h group, and 8.38 ± 1.51 points in the death within 30 days of hospitalization group, and the differences were statistically significant (*p* < 0.05). The average score was 2.74 ± 0.69 points in the outpatient treatment group, 4.19 ± 0.72 points in the emergency stay group, 5.40 ± 0.70 points in the specialist inpatient group, 8.71 ± 2.31 points in the ICU group, and 7.82 ± 1.66 points in the specialist unplanned transfer to ICU group, with the differences between the groups being statistically significant (*p* < 0.05). The average length of hospital stay for patients with a joint score within the range of 6–8 points was 10.86 ± 2.47 days, with a direct ICU admission rate of 22.00% and an unplanned ICU admission rate of 16.00%. Patients with a joint score >8 points had an average length of hospital stay of 27.05 ± 4.85 days, with a direct ICU admission rate of 66.67% and an unplanned ICU admission rate of 33.33%.

**Conclusion:**

Age and injury site are important high-risk indicators for trauma assessment, and using them in combination with the MEWS could improve the assessment of emergency patients with trauma, increasing the accuracy of pre-screening triage and reducing rescue time. Therefore, this joint scoring method might be worthy of clinical promotion and application.

## Introduction

Triage refers to the initial assessment of a patient's condition by the pre-screening triage nurse and the arrangement of appropriate medical treatment channels and treatment measures (1). Trauma is one of the important causes of human death, and it is currently the fourth cause of death among Chinese residents. The disability and fatality rate of people caused by trauma is still rising, and the success rate of trauma treatment in my country is much lower than that in developed countries (2). As more people have access to road transportation, road traffic accidents have increased, and trauma has become a major problem in emergency medical rescue. The Modified Early Warning Score (MEWS) system has been widely used in many countries because it is quick, easy, and practical (3–15). Scores are based on 5 items of body temperature, heart rate, consciousness, systolic blood pressure, and respiration. The higher the score, the more severe the disease, the higher the mortality rate and the ICU admission rate. MEWS ≥5 points often requires hospitalization; MEWS ≥9 points, the risk of death is significantly increased. However, the MEWS index only covers the most basic vital signs, and patients with severe trauma often have a combination of cranial, cervical, thoracic, abdominal, pelvic, and extremity injuries (16). Moreover, in clinical practice, age often determines the severity of the injury and the prognosis of a patient (3, 17). The present study was conducted to explore the establishment of a set of MEWS standards suitable for Chinese conditions. It is hoped that this will enable improvement in the accuracy of triage and the success rate of resuscitation of trauma patients in China, reduce the mortality and disability in these patients, and reduce the burden on affected families.

## Subjects and methods

### Subjects

Selected trauma patients in the emergency department of a tertiary hospital in Nantong City from June to December 2019 were the subjects for this study. Inclusion criteria were as follows: (1) trauma patients in the emergency department; (2) age ≥ 18 years; (3) complete medical records. Exclusion criteria were as follows: (1) patients who died before hospitalization or had undergone cardiopulmonary resuscitation; (2) patients with previous blood system diseases (such as anemia, leukemia, hemophilia, etc.).

### Methods

#### The MEWS combined with age and injury site scores

In clinical investigations (16–21), age and the injury site have been found to be high-risk indicators in determining the severity of trauma. In this study, age and the injury site were divided into high to low thresholds, and the patient's scores for these two factors were added to the MEWS, forming a new rating scale. The details can be seen in Table 1.

**Table 1:** MEWS combined with age and injury site score.

**Item**	**Score**						
	**3**	**2**	**1**	**0**	**1**	**2**	**3**
Heart rate (times/min)		<40	41–50	51–100	101–110	111–130	>130
Systolic blood pressure (mmHg)	<70	71–80	81–100	101–199		≥200	
Respiration rate (times/min)		<9		9–14	15–20	21–29	≥30
Body temperature (°C)		<35		35–38.4		≥38.5	
Consciousness				Clear	Be responsive to sound	Be responsive to pain	No response
Age (year)				18–39	40–59	≥60	
Injury site		The chest and abdomen	The maxillofacial		The four extremities	The pelvis, spine	The brain

#### Study methods

A retrospective study was used to collect data on emergency trauma patients from tertiary hospital in Nantong City in 2019. The included patients (according to the above criteria) were re-scored using the MEWS combined with age and injury site scores (hereinafter referred to as the “MEWS combined score” or “combined score”). The patients were grouped according to their different prognoses and outcomes. Based on the prognoses of patients, they were divided into the survival group and the death group; the death group was further divided into the death within 24 h group and the death within 30 days of hospitalization group. Based on the outcomes of patients, they were divided into the following five groups: outpatient treatment, emergency stay, specialist inpatient, ICU, and specialist unplanned transfer to ICU. The MEWS combined scores between different groups were compared, and the MEWS combined scores obtained by the patients were divided into intervals, and the average length of hospital stay, direct ICU admission rate, unplanned ICU admission rate, and emergency surgery rate of patients in different score ranges were statistically analyzed.

### Statistical methods

The SPSS 21.0 software package was used for all data processing, and descriptive statistics were carried out. The measurement data were expressed as means ± standard deviations ($\bar{x}$ ± s), and the categorical data were expressed as percentages (%). All continuous variables were tested for normality. The data were analyzed using the analysis of variance. The receiver operating characteristic (ROC) curve of the MEWS combined scores in predicting whether emergency trauma patients were hospitalized or not was drawn, and the area under the curve (AUROC) was calculated; *p* < 0.05 was considered statistically significant.

## Results

### General patient information

According to the criteria for inclusion and exclusion, 372 subjects were selected, including 223 males and 149 females, and the average age was 51.14 ± 17.34 years. There was no statistical difference in general patient data (*p* > 0.05) (Table 2).

**Table 2:** General demographic information.

**Age**	**Male**	**Female**
18–39	65	46
40–59	88	50
≥60	70	53

### The MEWS combined score

Among the 372 patients, the lowest combined score was 1 point, the highest was 16 points, and the average was 4.05 ± 1.82 points. There were 64 cases with a score of 0–2 points, accounting for 17.20%; 249 cases with a score of 3–5 points, accounting for 66.94%; 50 cases with a score of 6–8 points, accounting for 13.44%; and 9 cases with a score >8 points, accounting for 2.42%.

### The combined score in patients with different prognoses

Among the study subjects, 343 cases survived, 21 cases died within 24 h, and 8 cases died within 30 days of hospitalization. The combined score in the survival group was significantly lower than that in the death group, and the difference was statistically significant (*p* < 0.05) (Table 3).

**Table 3:** Results of MEWS score in patients with different outcomes ($\bar{X}\;\pm$ s, point).

**Item**	**Number of cases *(n)***	**The MEWS score**	***F***	***p***
Survival	343	3.68 ± 1.25		
Death within 24 h	21	8.33 ± 2.24	163.05	<0.001
Death within 30 days of hospitalization	8	8.38 ± 1.51		

### The combined score in patients with different outcomes

Among the 372 patients, the MEWS combined scores of the patients in the outpatient treatment group were the lowest, and the scores of the ICU inpatients were the highest. The combined scores of the five groups were significantly different (*p* < 0.05) (Table 4).

**Table 4:** Results of MEWS score in patients with different referrals ($\bar{X}\;\pm$ s, point).

**Item**	**Number of cases (*n*)**	**The MEWS score**	***F***	***P***
The outpatient	176	2.74 ± 0.69		
Kept observation in emergency department	79	4.19 ± 0.72		
The specialist hospitalization	89	5.40 ± 0.70	323.533	<0.001
ICU	17	8.71 ± 2.31		
The specialist unplanned transfer to ICU	11	7.82 ± 1.66		

### The comparison of the different combined score ranges

Among 372 patients, as the average hospital stay of the patients lengthened, the MEWS combined score gradually increased; when the score was >8 points, the average hospitalization rate of patients was as high as 27.05 ± 4.85 points. Similarly, the higher the patient's direct ICU occupancy rate and the higher the emergency surgery rate, the higher the combined score (Table 5).

**Table 5:** Comparison results of different MEWS score intervals.

		**The ICU admission rate (%)**		
**Item**	**The average length of hospital stay (Day)**	**The direct ICU admission rate**	**The unplanned transfer to ICU admission rate**	**The rate of emergency surgery (%)**
0–2 point	5.33 ± 1.27	0.00	0.00	0.00
3–5 point	9.18 ± 1.36	0.00	0.00	0.00
6–8 point	10.86 ± 2.47	22.00	16.00	28.00
>8 point	27.05 ± 4.85	66.67	33.33	77.78

### The predictive effect of the combined score with respect to patient admission

**Figure 1:**
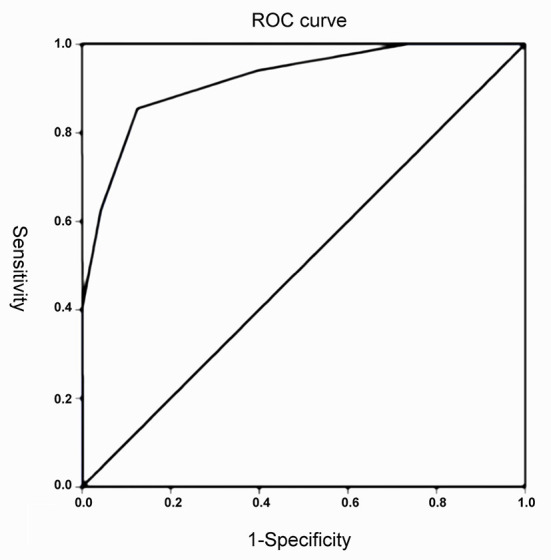
The receiver operating characteristic (ROC) curve of the Modified Early Warning Score (MEWS) combined score for predicting the hospitalization of patients.

The area under the curve of MEWS combined score ROC was 0.92 (95% CI: 0.890, 0.950), and the combined score for determining whether a patient needed hospitalization was of statistical significance (*p* < 0.05), as shown in Figure 1. The predictive effects of different thresholds when hospitalization was taken as the predictive target are shown in Table 6.

**Table 6:** Comparison of predictive effect of different cut-off points on admission in the patients.

**Item**	**Sensitivity (%)**	**1 - Specificity (%)**	**The youden index**
1 point	100	94.50	0.055
2 point	100	73.30	0.267
3 point	94.00	39.20	0.548
4 point	85.50	12.50	0.730
5 point	62.40	4.30	0.581

## Discussion

### The application of the combined score in assessing the pre-screening triage of emergency trauma patients

With the development of society and the increasing renewal of means of transportation, trauma has become a major problem in emergency rescue work. In the process of treating trauma patients, the success rate of treatment is often decreased due to factors such as the high occultity of the trauma itself, rapid progress, and severe illness (22). Assessing patients' injuries and making accurate and reasonable judgments also greatly affects the success rate of trauma treatment (23). Trauma triage is not only based on vital signs, but also needs to assess high-risk factors such as age, injury site, injury mechanism, etc. Because the mechanism of injury cannot be obtained quickly, emergency triage often fails to provide targeted assessment. In a study by a Hong Kong scholar (24), the use of MEWS score can help junior nurses to observe the condition of patients. In this study, the score of age and injury site was added on the basis of traditional MEWS. According to the results, MEWS combined age, injury and injury the site score can preliminarily judge whether the patient's injury is life threatening, and the triage nurses can use this score as an evaluation tool when evaluating the condition of the trauma patients.

### The application of the combined score in prognosis and outcome assessment of emergency patients with trauma

When the combined scores of the death within 24 h group and the death within 30 days of hospitalization group were compared, it was found that the combined score was higher in the former group than the latter. Of the patients in the five groups with different clinical outcomes, the patients in the outpatient treatment group had the lowest combined scores, while those in the direct ICU admission group had the highest scores. These results suggest that the combined score has some value in assessing the severity of the emergency in the trauma patient; this may assist the emergency resuscitation team in recognizing the patient's condition as quickly as possible and understanding the optimum time to implement resuscitation measures, as well as improving the success rate of patient resuscitation. In a study by Peng et al. (25), the MEWS was found to be of significant value when used to predict the severity of the condition of non-trauma patients; their findings were similar to those of the present study, where it was found that combining the two factors of injury site and age with the MEWS is very effective in predicting the severity of the condition of trauma patients. The results of the comparison of the different ranges of the combined scores showed that a higher combined score was correlated with a longer length of hospital stay and higher ICU admission rate and was also positively correlated with the unplanned transfer to ICU admission rate and the emergency surgery rate. In a study by Liu et al. (26) to predict in-hospital mortality and ICU transfer in infected and non-infected patients, the MEWS score was the best choice. It can be seen that the MEWS combined score has guiding significance for the injury of trauma patients after hospitalization, and can be used as a further research direction.

### The application of the combined score in the prediction of hospital admission of emergency patients with trauma

The merit of an evaluation system can be measured by plotting a ROC curve. In the present study, the AUROC was 0.92, indicating that the MEWS, combined with the age and injury site scores, has a high predictive value in determining hospital admission of emergency patients with trauma. The results were similar to the findings of Sun et al. (27), who added the two parameters of age and time of trauma to the MEWS, but the specificity of the evaluation in the present study was higher. It was found that the cutoff point of the MEWS for determining whether to admit an emergency trauma case was 5 points when Youden's index was calculated. When the MEWS, age, and injury site combined score in a trauma patient was ≥5 points, the hospitalization rate was higher, suggesting that the treatment pathway could be decided on and a reasonable treatment team allocated according to the situation when the patient arrives at the hospital.

The present study only investigated the specificity of the MEWS, age, and injury site combined score in the triage of trauma patients and the initial assessment of their condition. Although it could be inferred that a MEWS, age, and injury site combined score might be meaningful for guiding the assessment of injury in trauma patients after hospitalization, further investigations should be conducted to obtain more accurate scoring criteria for the follow-up assessment of hospitalized patients with trauma. These would be used in establishing a trauma scoring tool suitable for use in China so that the accuracy of triage and the success rate of treatment for trauma patients in China can be improved.

There are still many limitations in this research. Due to the impact of the COVID-19 epidemic in 2020, the study population selected for the present study was limited to patients who were hospitalized in 2019. At the same time, this current study only used the MEWS combined score for the preliminary assessment of the severity of the trauma in emergency patients and calculated the MEWS combined score to predict whether the trauma patient needed to be hospitalized. Furthermore, the specific criteria for the MEWS combined with the age and injury site scoring system still need to be explored and discussed. In addition, this study has not compared the MEWS combined score with the traditional MEWS and has not explored whether the MEWS combined score is more accurate and convenient than the traditional MEWS in clinical application. However, these issues can be explored in follow-up studies, as well as examining the assessment after trauma patients are hospitalized, in order to obtain more accurate scoring standards. This will provide a basis for establishing trauma scoring tools suitable for China's national conditions so as to improve the accuracy of triage of trauma patients in China and the success rate of treatment.

## Data availability statement

The original contributions presented in the study are included in the article/supplementary material, further inquiries can be directed to the corresponding author.

## Ethics statement

The study was conducted in accordance with the Declaration of Helsinki (as was revised in 2013). The study was approved by Ethics Committee of the Nantong First People's Hospital. Written informed consent was obtained from all participants.

## Author contributions

Y-FQ: conception, design of the research, analysis, and interpretation of the data. QL and D-FL: acquisition of data and statistical analysis. Y-QR: obtaining financing, writing of the manuscript, and critical revision of the manuscript for intellectual content. All authors have read and approved the final draft.

## Funding

The study was conducted in Nantong Municipal Health Commission Project and Guiding Scientific Research Fund of Nantong Science and Technology Bureau in 2019 (MSZ19175).

## Conflict of interest

The authors declare that the research was conducted in the absence of any commercial or financial relationships that could be construed as a potential conflict of interest.

## Publisher's note

All claims expressed in this article are solely those of the authors and do not necessarily represent those of their affiliated organizations, or those of the publisher, the editors and the reviewers. Any product that may be evaluated in this article, or claim that may be made by its manufacturer, is not guaranteed or endorsed by the publisher.
